# Development and validation of web‐based prognostic nomograms for massive hepatocellular carcinoma (≥10 cm): A retrospective study based on the SEER database

**DOI:** 10.1002/cam4.6003

**Published:** 2023-04-27

**Authors:** Guizhong Huang, Qiaohong Lin, Pengfei Yin, Kai Mao, Jianlong Zhang

**Affiliations:** ^1^ Department of Hepatobiliary Surgery Sun Yat‐Sen Memorial Hospital, Sun Yat‐Sen University Guangzhou China; ^2^ Department of Pancreatobiliary Surgery, State Key Laboratory of Oncology in South China, Collaborative Innovation Center for Cancer Medicine Sun Yat‐Sen University Cancer Center Guangzhou P. R. China; ^3^ Department of Head and Neck Surgery, State Key Laboratory of Oncology in South China, Guangdong Key Laboratory of Nasopharyngeal Carcinoma Diagnosis and Therapy Sun Yat‐Sen University Cancer Center Guangzhou P. R. China

**Keywords:** cancer‐specific survival, hepatocellular carcinoma, nomogram, overall survival, SEER database

## Abstract

**Background and Aims:**

Massive hepatocellular carcinoma (MHCC, a maximum tumor size of at least 10 cm) tends to have a poor prognosis. Therefore, this study aims to construct and validate prognostic nomograms for MHCC.

**Methods:**

Clinic data of 1292 MHCC patients between 2010 and 2015 were got from the surveillance, epidemiology, and end results (SEER) cancer registration database. The whole set was separated into the training and validation sets at a ratio of 2:1 randomly. Variables, significantly associated with cancer‐specific (CSS) and overall survival (OS) of MHCC were figured out by multivariate Cox regression analysis and were taken to develop nomograms. The concordance index (C‐index), calibration curve, and decision curve analysis (DCA) were taken to validate the predictive abilities and accuracy of the nomograms.

**Results:**

Race, alpha‐fetoprotein (AFP), grade, combined summary stage, and surgery were identified as independent factors of CSS, and fibrosis score, AFP, grade, combined summary stage, and surgery significantly correlated with OS in the training cohort. They then were taken to construct prognostic nomograms. The constructed model for predicting CSS exhibited satisfactory performance with a C‐index of 0.727 (95% CI: 0.746–0.708) in the training group and 0. 672 (95% CI: 0.703–0.641) in the validation group. Besides, the model for predicting OS of MHCC also showed strong performance both in the training group (C‐index: 0.722, 95% CI: 0.741–0.704) and the validation (C‐index: 0.667, 95% CI: 0.696–0.638) group. All calibration curves and decision curves performed satisfactory predictive accuracy and clinic application values of the nomograms.

**Conclusion:**

The web‐based nomograms for CSS and OS of MHCC were developed and validated in this study, which prospectively could be tested and may serve as additional tools to assess patient's individualized prognosis and make precise therapeutic selection to improve the poor outcome of MHCC.

## 1 INTRODUCTION

Hepatocellular carcinoma (HCC) is one of the most common cancers worldwide and the leading cause of tumor‐related mortality in primary liver cancer.^1^ The overall survival (OS) of HCC is impacted by several factors such as tumor number, vascular invasion, treatment options feasible, etc.^2^, ^3^, ^4^, ^5^ But tumor size is one of the most notable prognostic indicators for HCC.^2^, ^6^, ^7^ Patients with huge tumors tend to have an advanced stage and thus leading to poorer prognoses.^7^ Even for a patient receiving tumor resection, a huge tumor is associated with early recurrence within 2 years after primary surgery.^7^, ^8^ Due to the increasing improvement in public awareness of physical examinations, many HCC patients are now detected and diagnosed at an early stage. However, there is still a considerable proportion of patients who present with large tumors and advanced stages due to a lack of awareness of the importance of health maintenance, especially in China.^9^

Massive HCC (MHCC), one of the subtypes of HCC, usually refers to the notion that a patient suffers a maximum tumor size of at least 10 cm.^10^, ^11^ It is reported that MHCC accounts for 5.7% of all HCC types.^12^ Such subtype is often accompanied by macrovascular invasion, satellite nodules, metastases, and other aggressive characteristics. Besides, MHCC patients usually accept extensive resection, which increases the risk of postoperative liver failure and perioperative mortality. Compared with a tumor <10 cm, the prognosis of a huge tumor after surgery was dissatisfactory [the 5‐year OS rate of approximately 30%].^13^, ^14^ Remarkably, some studies suggested that patient with MHCC who was unable to accept surgery had a worse 5‐year OS rate of <20%.^15^, ^16^, ^17^, ^18^ There was still a lack of enough approaches and studies to discriminate high‐risk patients in the MHCC cohort. Under the consciousness of precision medicine, how to effectively intervene in the progression of MHCC deserves clinicians' attention and efforts. Therefore, it is necessary to clarify those risk factors that can independently impact the long‐term outcome of MHCC and to take corresponding measures to improve the poor prognosis for MHCC patients.

In this study, we aim to figure out those significantly independent indicators of cancer‐specific survival (CSS) and OS for MHCC and to construct web‐based prognostic nomograms by using data from the surveillance, epidemiology, and end results (SEER) cancer registration database. We think the constructed predictive models will effectively and conveniently help clinicians provide MHCC patients with appropriately personalized interventions.

## 2 METHODS AND MATERIALS

### 2.1 Patient selection

The medical data of a total of 104,173 HCC cases between 2010 and 2015 were extracted from the SEER plus database [18 registries (2000–2018)]. The inclusion criteria in this study were as follows: (I) adult patients (age ≥18 years old); (II) positive cytology or histology evidence of HCC (ICD‐O‐38170/3 to 8175/3); (III) tumor size ≥10 cm; (IV) survival time ≥1 month. Cases having inadequate clinic data were excluded. Limited by lagging in data updates of the SEER database, we excluded the patients from before 2010 and from 2015 onwards to reduce calculative bias caused by vast missing data of some variables.

### 2.2 Medical variables

Medical data comprised baseline demographics [race (White, Black, or Other), sex, age at diagnosis (≤65 years old or >65 years old), year of diagnosis (2010–2011, 2012–2013, or 2014–2015), marital status (married or other), preoperative alpha‐ fetoprotein (AFP) status (negative/normal or positive/elevated), and fibrosis score (Ishak 0–4, Ishak 5–6 or unknown)], tumor‐related characteristics [tumor size (10–20 cm or >20 cm), grade (grade I/II or III/IV), stage according to the 7th AJCC‐TNM staging system, combined summary stage (localized, regional or distant)], therapeutic strategies (with or without surgery, with or without radiotherapy, and with or without chemotherapy), survival condition (survival months and vital status). Notably, as for histological grade of differentiation, grade I/II refers to a well or a moderately differentiated tumor while grade III/IV means a poorly differentiated or an undifferentiated tumor.

### 2.3 Statistical analysis

The medical data of all cases were extracted by the SEER *Stat 8.3.9 software. IBM SPSS Statistics 25.0, R 4.1.3 (www.R‐project.org), and MedCalc 19.0 were used for all statistical analyses. The whole set was then divided into the training and validation cohorts at a ratio of 2:1 randomly. Continuous variables are summarized as “mean ± SD” and their differences between groups were compared by the Mann–Whitney *U* test. Discontinuous variables are expressed as numbers and percentages. The chi‐square test or Fisher's exact test were utilized to explore differences in discontinuous variables between groups. The significantly independent factors of CSS and OS, identified by conducting Cox multi‐factor analyses, were utilized to construct prognostic nomograms. The concordance index (C‐index), calibration curve, and decision curve analysis (DCA) were used to validate the predictive abilities and accuracy; and then, the validation cohort was used to further evaluate the reliability and accuracy of the constructed nomograms. To elaborate on the prognostic differences between subgroups, Kaplan–Meier survival curve analysis and log‐rank test were performed. At last, two web applications based on the constructed nomograms for CSS and OS of MHCC were developed and deployed. A *p* < 0.05 in a two‐tailed test was deemed to have statistical significance.

## 3 RESULTS

### 3.1 General characteristics

A total of 1292 MHCC eligible patients were recruited for further analysis. 49.1% of the whole cohort were diagnosed at age over 65 years old. Besides, majorities of them were male (78.6%), White (64%), and married (59.2%). Notably, 71.9% of the patients presented a positive or elevated status of AFP before treatment, the average tumor size was 14.7 cm, and 30.8% of the tumors were grade III/IV. Approximately one‐third of the patients underwent surgery, while 176 (13.6%) patients accepted radiotherapy and 620 (48.0%) patients received chemotherapy. No statistical difference was found between the training and validation cohorts, and their baseline clinical information was exhibited in Table 1.

**TABLE 1:** Basic clinicopathological characteristics in the total, training, and validation sets.

Variables	Total set (*n* = 1292)	Training set (*n* = 861)	Validation set (*n* = 431)	*p*‐value
Age (years)	65.2 ± 12.6	65 ± 12.3	65.5 ± 13.2	0.269
Sex				
Female	277 (21.4%)	184 (21.4%)	93 (21.6%)	0.943
Male	1015 (78.6%)	677 (78.6%)	338 (78.4%)	
Race				
White	827 (64%)	542 (63%)	285 (66.1%)	0.518
Black	179 (13.9%)	124 (14.4%)	55 (12.8%)	
Other^a^	286 (22.1%)	195 (22.6%)	91 (21.1%)	
Marital status				
Married	765 (59.2%)	517 (60%)	248 (57.5%)	0.401
Other^b^	527 (40.8%)	344 (40%)	183 (42.5%)	
Year of diagnosis				
2010–2011	420 (32.5%)	283 (32.9%)	137 (31.8%)	0.826
2012–2013	438 (33.9%)	287 (33.3%)	151 (35%)	
2014–2015	434 (33.6%)	291 (33.8%)	143 (33.2%)	
Fibrosis score				
Ishak 0–4	191 (14.8%)	129 (15%)	62 (14.4%)	0.933
Ishak 5–6	144 (11.1%)	635 (73.8%)	322 (74.7%)	
Unknown	957 (74.1%)	97 (11.2%)	47 (10.9%)	
AFP status				
Negative/normal	363 (28.1%)	238 (27.6%)	125 (29%)	0.646
Positive/elevated	929 (71.9%)	623 (72.4%)	306 (71%)	
Tumor size (cm)	14.7 ± 9.7	14.7 ± 10	14.8 ± 9.1	0.834
Grade				
I/II	894 (69.2%)	599 (69.6%)	295 (68.4%)	0.702
III/IV	398 (30.8%)	262 (30.4%)	136 (31.6%)	
Combined summary stage				
Localized	490 (37.9%)	330 (38.3%)	160 (37.1%)	0.608
Regional	526 (40.7%)	354 (41.1%)	172 (39.9%)	
Distant	276 (21.4%)	177 (20.6%)	99 (23%)	
T stage				
T 1–2	518 (40.1%)	337 (39.1%)	181 (42%)	0.336
T 3–4	774 (59.9%)	524 (60.9%)	250 (58%)	
N stage				
N 0	1143 (88.5%)	766 (89%)	377 (87.5%)	0.46
N 1	149 (11.5%)	95 (11%)	54 (12.5%)	
M Stage				
M 0	1045 (80.9%)	697 (81%)	348 (80.7%)	0.94
M 1	247 (19.1%)	164 (19%)	83 (19.3%)	
Surgery				
No	890 (68.9%)	581 (67.5%)	309 (71.7%)	0.127
Yes	402 (31.1%)	280 (32.5%)	122 (28.3%)	
Chemotherapy				
No	672 (52%)	445 (51.7%)	227 (52.7%)	0.768
Yes	620 (48%)	416 (48.3%)	204 (47.3%)	
Radiotherapy				
No	1116 (86.4%)	739 (85.8%)	377 (87.5%)	0.44
Yes	176 (13.6%)	122 (14.2%)	54 (12.5%)	
Vital status				
Alive	175 (13.5%)	120 (13.9%)	55 (12.8%)	0.605
Dead	1117 (86.5%)	741 (86.1%)	376 (87.2%)	
Survival months [median (range)]	8 (1–107)	8 (1–107)	8 (1–106)	0.189

Abbreviation: AFP, alpha fetoprotein.

^a^ Other includes Asian/Pacific Islander, American Indian/Alaskan Native.

^b^ Other includes single, unmarried, separated, divorced, widowed, domestic partner, and unknown.

### 3.2 Survival analyses for CSS and OS

The median survival time of the total set was 8 months (ranges from 1 to 107 months). The 1‐, 3‐, and 5‐year CSS rate of the whole set were 42.9%, 23%, and 17.6% while the 1‐, 3‐, and 5‐year OS rate were 39.5%, 19.8%, and 13.8%, respectively. According to the results of the multivariate analyses in the training cohort, AFP, grade, combined summary stage, and surgery were identified as independent factors of both CSS and OS for MHCC. Besides, race was one of the risk factors for CSS, and fibrosis score was a significant statistical indicator for OS. Detailed information was shown in Tables 2 and 3.

**TABLE 2:** Cox regression analyses for cancer‐specific survival in the training set.

Variables	Univariate analysis		Multivariate analysis	
	HR (95% CI)	*p*‐value	HR (95% CI)	*p*‐value
Age (years)				
≤65	Ref.	–	–	–
>65	0.96 (0.825–1.117)	0.597	–	–
Sex				
Female	Ref.	–	–	–
Male	1.35 (1.116–1.634)	0.002*	–	–
Race				
White	Ref.	–	Ref.	–
Black	1.202 (0.968–1.492)	0.096	1.012 (0.813–1.259)	0.917
Other^a^	0.745 (0.613–0.904)	0.003*	0.766 (0.63–0.933)	0.008*
Marital status				
Married	Ref.	–	–	–
Other^b^	1.159 (0.993–1.353)	0.061	––	–
Year of diagnosis				
2010–2011	Ref.	–	–	–
2012–2013	1.161 (0.966–1.396)	0.112	–	–
2014–2015	1.038 (0.857–1.256)	0.704	–	–
Fibrosis score				
Ishak 0–4	Ref.	–	–	–
Ishak 5–6	1.865 (1.475–2.358)	<0.001***	–	–
Unknown	1.793 (1.305–2.463)	<0.001***		
AFP status				
Negative/normal	Ref.	–	Ref.	–
Positive/elevated	1.8 (1.505–2.152)	<0.001***	1.651 (1.369–1.991)	<0.001***
Tumor size (cm)				
10–20	Ref.	–	–	–
>20	1.452 (1.033–2.042)	0.032*	–	–
Grade				
I/II	Ref.	–	Ref.	–
III/IV	1.633 (1.387–1.922)	<0.001***	1.712 (1.437–2.039)	<0.001***
Combined summary stage				
Localized	Ref.	–	Ref.	–
Regional	1.948 (1.629–2.329)	<0.001***	1.484 (1.236–1.78)	<0.001***
Distant	3.516 (2.836–4.359)	<0.001***	2.264 (1.816–2.824)	<0.001***
T stage				
T 1–2	Ref.	–	–	–
T 3–4	1.888 (1.604–2.222)	<0.001***	–	–
N stage				
N 0	Ref.	–	–	–
N 1	1.881 (1.49–2.375)	<0.001***	–	–
M stage				
M 0	Ref.	–	–	–
M 1	2.355 (1.951–2.843)	<0.001***	–	–
Surgery				
No	Ref.	–	Ref.	–
Yes	0.326 (0.272–0.391)	<0.001***	0.328 (0.271–0.397)	<0.001***
Chemotherapy				
No	Ref.	–	–	–
Yes	1.137 (0.976–1.324)	0.1	–	–
Radiotherapy				
No	Ref.	–	–	–
Yes	0.994 (0.805–1.227)	0.954	–	–

Abbreviation: AFP, alpha fetoprotein.

^a^ Other includes Asian/Pacific Islander, American Indian/Alaskan Native.

^b^ Other includes single, unmarried, separated, divorced, widowed, domestic partner, and unknown.

* *p* < 0.05

*** *p* < 0.001.

**TABLE 3:** Cox regression analyses for overall survival in the training set.

Variables	Univariate analysis		Multivariate analysis	
	HR (95% CI)	*p*‐value	HR (95% CI)	*p*‐value
Age (years)				
≤65	Ref.	–	–	–
>65	0.974 (0.844–1.125)	0.723	–	–
Sex				
Female	Ref.	–	–	–
Male	1.401 (1.167–1.682)	<0.001***	–	–
Race				
White	Ref.	–	–	–
Black	1.218 (0.992–1.496)	0.06	–	–
Other^a^	0.777 (0.648–0.932)	0.007*	–	–
Marital status				
Married	Ref.	–	–	–
Other^b^	1.162 (1.004–1.346)	0.044*	–	–
Year of diagnosis				
2010–2011	Ref.	–	–	–
2012–2013	1.162 (0.974–1.385)	0.095	–	–
2014–2015	1.085 (0.906–1.301)	0.376	–	–
Fibrosis score				
Ishak 0–4	Ref.	–	Ref.	–
Ishak 5–6	1.936 (1.544–2.426)	<0.001***	1.426 (1.131–1.798)	0.003*
Unknown	2.042 (1.516–2.749)	<0.001***	1.507 (1.114–2.037)	0.008*
AFP status				
Negative/normal	Ref.	–	Ref.	–
Positive/elevated	1.698 (1.436–2.007)	<0.001***	1.562 (1.312–1.859)	<0.001***
Tumor size (cm)				
10–20	Ref.	–	–	–
>20	1.382 (0.992––1.923)	0.056	–	–
Grade				
I/II	Ref.	–	Ref.	–
III/IV	1.606 (1.375–1.876)	<0.001***	1.613 (1.365–1.907)	<0.001***
Combined summary stage				
Localized	Ref.	–	Ref.	–
Regional	1.852 (1.566–2.192)	<0.001***	1.424 (1.2–1.69)	<0.001***
Distant	3.282 (2.675–4.027)	<0.001***	2.178 (1.764–2.688)	<0.001***
T stage				
T 1–2	Ref.	–	–	–
T 3–4	1.845 (1.582–2.152)	<0.001***	–	–
N stage				
N 0	Ref.	–	–	–
N 1	1.871 (1.498–2.337)	<0.001***	–	–
M stage				
M 0	Ref.	–	–	–
M 1	2.287 (1.908–2.74)	<0.001***	–	–
Surgery				
No	Ref.	–	Ref.	–
Yes	0.333 (0.281–0.395)	<0.001***	0.348 (0.289–0.418)	<0.001***
Chemotherapy				
No	Ref.	–	–	–
Yes	1.104 (0.955–1.276)	0.182	–	–
Radiotherapy				
No	Ref.	–	–	–
Yes	0.958 (0.782–1.174)	0.681		

Abbreviation: AFP, alpha fetoprotein.

^a^ Other includes Asian/Pacific Islander, American Indian/Alaskan Native.

^b^ Other includes single, unmarried, separated, divorced, widowed, domestic partner, and unknown.

* *p* < 0.05

*** *p* < 0.001.

### 3.3 Construction and validation of nomogram predicting CSS

**FIGURE 1:**
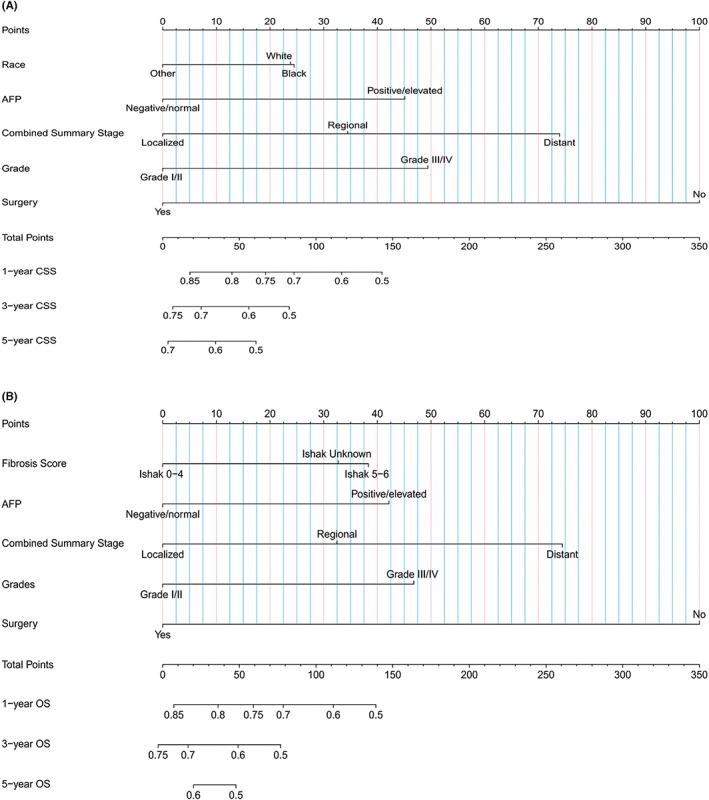
Nomograms for predicting CSS (A) and OS (B) of MHCC. AFP, alpha fetoprotein; CSS, cancer‐specific survival; MHCC, massive hepatocellular carcinoma; OS, overall survival.

**FIGURE 2:**
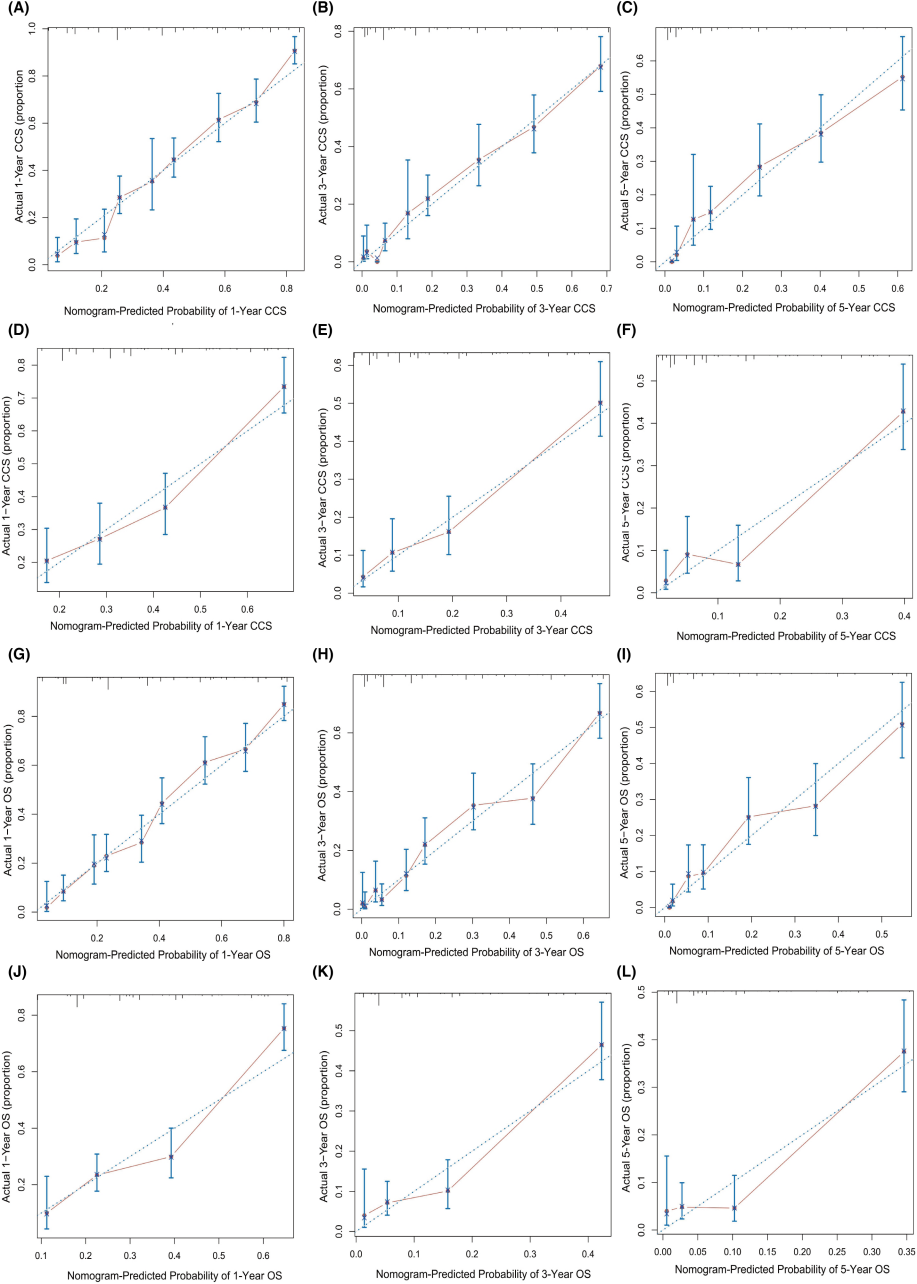
Calibration curves of the constructed nomograms. The predictive models for CSS of MHCC predicted 1‐ (A for the training cohort and D for the validation cohort), 3‐ (B for the training cohort and E for the validation cohort), and 5‐year (C for the training cohort and F for the validation cohort) CSS. The nomogram for OS of MHCC predicted 1‐ (G for the training cohort and J for the validation cohort), 3‐ (H for the training cohort and K for the validation cohort), and 5‐year (I for the training cohort and L for the validation cohort) OS. The actual 1‐, 3‐, and 5‐year CSS/OS was plotted on the y‐axis, and the x‐axis exhibited the model‐estimated CSS/OS. CSS, cancer‐specific survival; MHCC, massive hepatocellular carcinoma; OS, overall survival.

**FIGURE 3:**
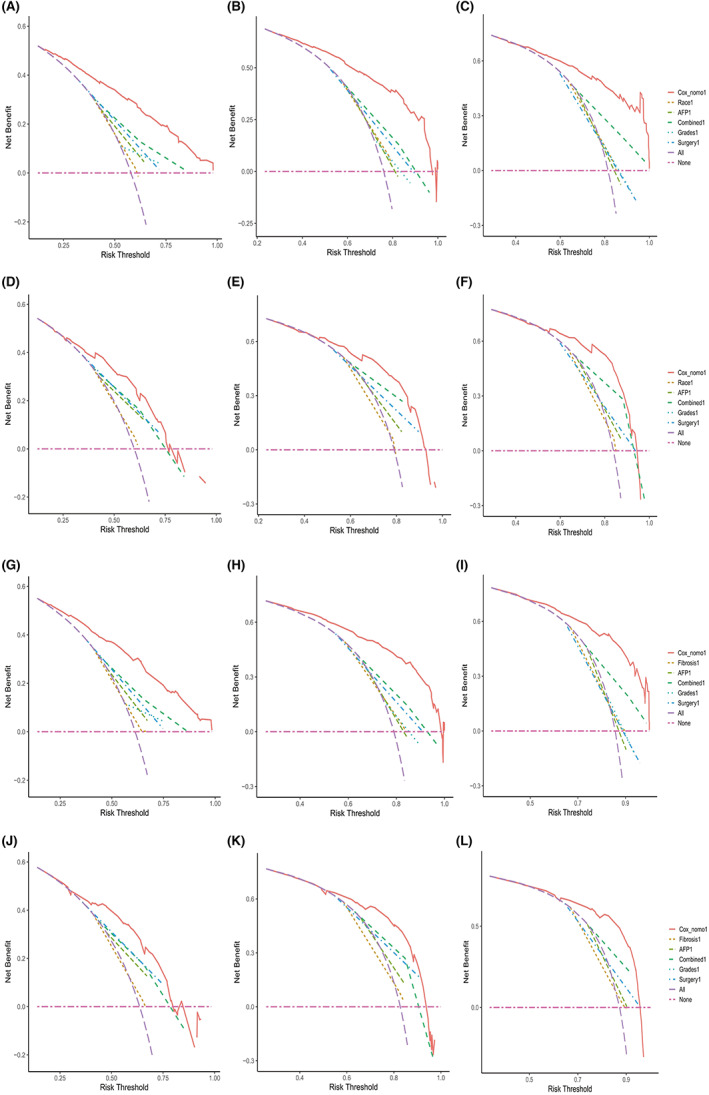
Decision curve analyses (DCA) of the nomograms for CSS and OS of MHCC. DCA was taken to compare the clinic net benefit between the model and other significant independent factors of CSS/OS in predicting 1‐ (CSS: A for training set and D for validation set; OS: G for training set and J for validation set), 3‐ (CSS: B for training set and E for validation set; OS: H for training set and K for validation set), and 5‐year (CSS: C for training set and F for validation set; OS: I for training set and L for validation set) CSS and OS rates for MHCC. On the decision curve analysis, the horizontal line represents the assumption that no patient will die, and the oblique line represents the assumption that all patients will die. CSS, cancer‐specific survival; MHCC, massive hepatocellular carcinoma; OS, overall survival.

A nomogram predicting CSS for MHCC was developed and then deployed the model into an open‐access online calculator based on the outcomes of the multivariate analysis (https://sunyatsenmemorialhosipital.shinyapps.io/CSS_MHCC/) Figure 1A. The model showed great performance for predicting CSS in the training group (C‐index: 0.727, 95% CI: 0.746–0.708) and the validation groups (C‐index: 0. 672, 95% CI: 0.703–0.641). Besides, the calibration curves also exhibited the great predictive ability of the developed model as they were highly consistent with the actual observation Figure 2A–F. Moreover, compared with using sole risk factors to predict CSS, the decision curves for the constructed nomogram revealed the greatest net benefit Figure 3A–F.

**FIGURE 4:**
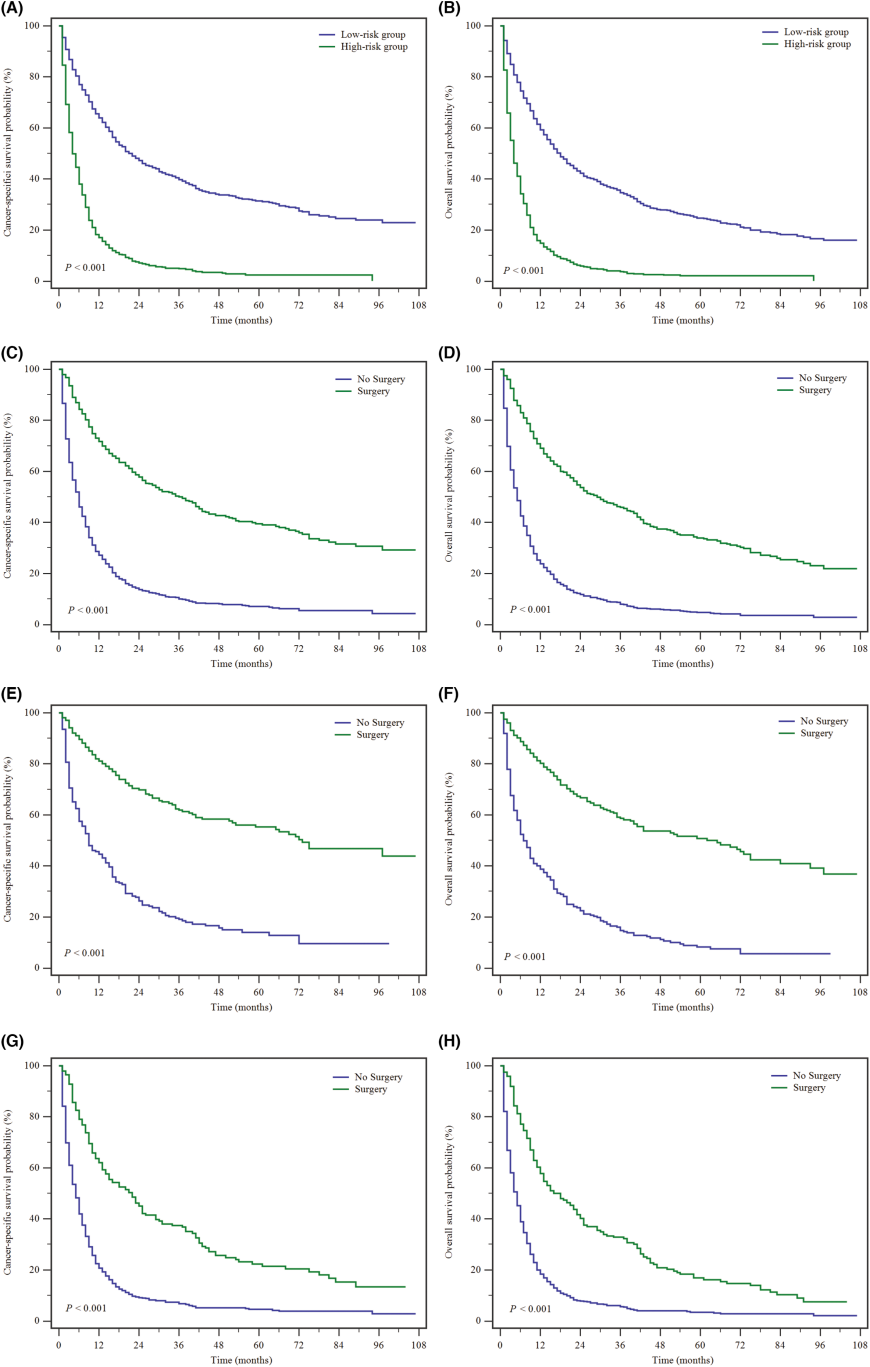
Kaplan–Meier survival analyses to estimate CSS and OS for MHCC stratified by the nomograms (A for CSS, and B for OS) in the whole set, and by surgery in the total set (C for CSS, and D for OS), in the TNM I‐II stage set (E for CSS, and F for OS), and in the TNM III‐IV stage set (G for CSS, and H for OS).

Based on the above dynamic nomogram, the total cohort was then separated into high‐risk and low‐risk groups by the median score of 169 after calculating the sum risk scores. The median CSS values were 21 and 4 months in the low‐risk and high‐risk groups, respectively. And the 5‐year CSS rate in the low‐risk group of the whole cohort was substantially better than that in the high‐risk group (31.4% vs. 2.3%, *p* < 0.001) (Figure 4A).

### 3.4 Construction and validation of nomogram predicting OS

We also developed a nomogram of predicting OS for MHCC according to the outcomes of the multi‐factors analysis. We then constructed a web‐based calculator to facilitate the use of the nomogram (https://sunyatsenmemorialhosipital.shinyapps.io/OSMHCC/) (Figure 1B).

The nomogram also showed satisfactory performance for predicting OS with a C‐index of 0.722 (95% CI: 0.741–0.704) in the training group and 0.667 (95% CI: 0.696–0.638) in the validation group. Besides, the calibration curves for the probability of 1‐, 3‐, and 5‐year OS were also highly consistent with the actual observation (Figure 2G–L). Moreover, decision curve analyses for the constructed nomogram revealed great clinic application values (Figure 3G–L).

According to the above online nomogram, the total cohort was also separated into high‐risk and low‐risk groups by the median score of 175 after calculating the sum risk scores. The 5‐year OS rate in the high‐risk set was poorer than that in the low‐risk set (2% vs. 24.7%, *p* < 0.001) (Figure 4B) and the median OS values were 18 and 4 months in the low‐risk and high‐risk sets, respectively.

### 3.5 Subgroup analyses

The Kaplan–Meier curves suggested that MHCC patients accepting surgery could get better long‐term outcomes. The 5‐year CSS rate was 39.4% in the surgery set and 7% in the no‐surgery set (*p* < 0.001). In addition, the OS rate at 5 year in the surgery set was better than that in the no‐surgery set (33.9% vs. 4.6%, *p* < 0.001) (Figure 4C,D).

Moreover, in the early‐stage (TNM stage I/II) cohort, the median CSS values in the surgery group and the no surgery group were 73 and 9 months, respectively (*p* < 0.001). And the median OS values were 62 and 7 months in the surgery and no‐surgery groups, respectively (*p* < 0.001). While in the advanced stage (TNM stage III/IV) cohort, the median CSS and OS values in the surgery group were 22 and 16 months, respectively. But the median CSS value in the no‐surgery group was 5 months, and the median OS value was 5 months (Figure 4E–H).

## 4 DISCUSSION

In this study, we found that the prognosis of MHCC was associated with several independent factors such as preoperative serum AFP status, grade, surgery, etc. According to the above results, we constructed two nomograms harvesting satisfactory predictive abilities to evaluate CSS and OS rates at 1‐, 3‐, and 5‐year for MHCC. It may be helpful to assist in making a precisely prognostic evaluation, therapeutic selection, and individual follow‐up strategy. Although some predictive models evaluating the prognosis of MHCC exist, they were mainly developed based on cases from a single race/region, and did not discuss whether other treatment strategies could impact the prognosis of MHCC or not.^19^, ^20^ The SEER database, an authoritative source, provides many cross‐race cases with detailed information for studies. Based on data from the database, we constructed and validated the prognostic models for MHCC with more persuasiveness. By clicking the corresponding variables' status on the online operation interface we provided, clinicians can effectively and precisely evaluate the prognosis of MHCC, regardless of patient from White or non‐White population.

Nowadays, several therapeutic choices can be selected for HCC, including surgery, radiofrequency ablation, anhydrous alcohol injection, and transhepatic artery chemotherapy and embolization (TACE), etc.^21^, ^22^, ^23^, ^24^ But surgery is usually taken as the first‐line curative modality for either resectable HCC or as a bridge for future salvage liver transplantation. Certainly, a complete resection of the tumor contributes to improving the poor prognosis of MHCC. Zhu et al^13^ retrospectively analyzed the efficacy of surgery for HCC with a tumor larger than 10 cm. Their result showed that the MHCC group could get a 5‐year OS rate of 30.3% after primary resection. Another study reported that surgery could contribute to a 5‐year OS rate of 44.1% and a 5‐year disease‐free survival (DFS) rate of 42.2% for MHCC after resection.^25^ However, compared with those who accepted surgery, patients receiving nonsurgical treatment have unsatisfactory long‐term outcomes. After comparing the efficacy difference of the prognosis of MHCC between surgery and TACE, Min reported that the surgery group showed a higher 5‐year OS rate than the TACE group (39.8% vs. 9.7%, *p* < 0.001).^16^ The same results could be found in other studies.^21^, ^22^ In our study, multivariate Cox regression analyses figured out that surgery can impact both OS and CSS of MHCC to some extent. Therefore, it was included in our constructed nomograms. In our study, the median CSS and OS values in the no‐surgery group were 6 and 5 months, respectively. However, the group successfully accepting surgery had higher 5‐year CSS and OS rates (*p* < 0.001). Even patients at advanced‐stage can improve their prognosis by getting surgery (the median CSS values: 22 vs. 5 months, *p* < 0.001). Those results reflect that the patients can gain better prognosis by accepting surgery, if eligible. But limited by the data provided by the SEER database selected, we did not further discuss the specific influences of different surgery methods on the prognosis of MHCC. What's more, we deeply analyzed the influences of chemotherapy and radiotherapy on the prognosis of MHCC by Cox regression analysis. Surprisingly, the results showed that those treatment methods did not significantly impact the long‐term outcomes for MHCC patients.

However, surgery for MHCC often means extensive liver resection which may lead to insufficient residual liver volume, increase the risk of postoperative complications, and even death. Some studies reported the morbidity and mortality rates of MHCC after major resection ranging from 10.9% to 43.6% and 4.2% to 18.1%, respectively.^26^, ^27^, ^28^, ^29^, ^30^ A retrospective study of 525 huge HCSS reported a postoperative complication rate of 26.4% and a 30‐day mortality rate of 2.7%.^28^ Even some unconventional procedures can still yield safe short‐term outcomes. Jia et al^31^ evaluated the safety of anatomic trisegmentectomy for huge HCC of the right liver. In their study, 13 patients accepted anatomic trisegmentectomy with complete resection of a tumor but without suffering perioperative morbidity and mortality. Even performed under laparoscopy and accompanied with a higher Iwate difficulty score, patients can still get safe outcomes that 90‐day mortality of <1%.^32^ Common postoperative complications include ascites, bile leakage, intra‐abdominal bleeding, infection, and liver failure. Fei et al^33^ constructed a model predicting postoperative liver failure for huge HCC based on preoperative CT radiomics. They figured out that the excision extension, the model for end‐stage liver disease (MELD) score, and the radiomics score (Radscore) were significantly risking indicators for the postoperative severe liver failure after major resection. By their model, a surgeon can take enough perioperative prevention measures to decrease the risk of postoperative liver failure. Therefore, although extensive hepatectomy may make MHCC patients vulnerable to various complications and even death, the surgeon can still perform a safe surgery through preoperative comprehensive assessments, intra‐operative precise resection, and enhanced recovery after surgery.

Other variables in our models include histological grade, preoperative serum AFP status, etc. Numerous studies showed that they all were associated with the prognosis of HCC.^34^, ^35^, ^36^, ^37^ Chen et al^20^ figured out that both cirrhosis and AFP were significantly independent factors of OS for HCC. Meanwhile, compared with the non‐MHCC set, the MHCC set had a higher level of preoperative AFP.^10^ But we did not further discuss whether the discrepancy of elevated AFP could impact patients' prognoses to a different extent or not, due to a lack of concrete values of AFP. Notably, the results of our study suggested that the further increased diameter of the tumor did not exert a significant impact on the prognosis of MHCC. The main reason may be that we just divide the whole set into a 10–20 cm group and a >20 cm group which could be less sensitive for predicting prognoses of MHCC. The pathohistological differentiation of a tumor focus matters a lot to the appraisal of long‐term outcomes. A poorly differentiated tumor combined with an elevated AFP level significantly impacts the risk of early recurrence for HCC after surgery.^38^ Strikingly, HCC patients along with cirrhosis may endure unsatisfactory survival after orthotopic liver transplantation if diagnosed with a higher histological grade.^39^ Therefore, no need to hesitation for clinicians to consider all‐around variables that would impact the prognoses of MHCC while making individualized strategies.

Several limitations exist in this study. First, even if the data utilized in this study were extracted from the public database that provided a sea of cross‐race HCC cases, the developed nomograms still need further external validation to test their predictive accuracy and performance. Second, limited by the specific variables provided by the SEER database, we did not discuss the associations between specific clinical variables such as etiology, liver function status, vascular invasion, and prognoses of MHCC. Besides, numerous staging systems have been developed and widely employed to evaluate long‐term outcome for HCC including the Barcelona Clinic Liver Cancer (BCLC) system and Cancer of the Liver Italian Program (CLIP).^22^ However, limited by the SEER database accessibility, we did not conduct an in‐depth discussion about the roles of other tumor staging systems on the prognostic evaluation of MHCC compared with our prognostic models. The online predictive models above can serve as convenient but useful tools to help clinicians quickly evaluate the concrete prognosis and make more individualized therapeutic strategies for MHCC patients to get better long‐term outcomes.

## 5 CONCLUSION

In summary, we constructed two web‐based predictive nomograms for clinical surgeons to precisely evaluate the prognosis of MHCC. Both of the models exhibited satisfactory predictive abilities and clinical application values.

## AUTHOR CONTRIBUTIONS

**Guizhong Huang:** Conceptualization (equal); data curation (equal); formal analysis (lead); investigation (lead); methodology (equal); project administration (equal); software (equal); validation (equal); visualization (lead); writing – original draft (lead); writing – review and editing (equal). **Qiaohong Lin:** Data curation (equal); formal analysis (equal); writing – original draft (equal); writing – review and editing (equal). **Pengfei Yin:** Data curation (equal); formal analysis (equal); writing – original draft (equal). **Kai Mao:** Conceptualization (equal); supervision (equal); writing – original draft (equal); writing – review and editing (equal). **Jianlong Zhang:** Conceptualization (equal); project administration (lead); resources (lead); supervision (equal); writing – original draft (equal); writing – review and editing (equal).

## FUNDING INFORMATION

This research received no external funding.

## CONFLICT OF INTEREST STATEMENT

The authors have no conflicts of interest to declare.

## ETHICAL APPROVAL STATEMENT

As the study consisted of the retrorespective analysis of anonymous data according to the local ethics committee, a special approval is generally not required.

## Data Availability

The data of this study are available in the SEER database (https://seer.cancer.gov/).

## References

[1] McGlynn KA , Petrick JL , El‐Serag HB . Epidemiology of hepatocellular carcinoma. Hepatology. 2021;73(Suppl 1):4‐13. 10.1002/hep.31288 PMC7577946 32319693

[2] Huang WJ , Jeng YM , Lai HS , Sheu FYB , Lai PL , Yuan RH . Tumor size is a major determinant of prognosis of resected stage I hepatocellular carcinoma. Langenbecks Arch Surg. 2015;400(6):725‐734. 26250145 10.1007/s00423-015-1329-4

[3] Cai M , Huang W , Huang J , et al. Transarterial chemoembolization combined with lenvatinib plus PD‐1 inhibitor for advanced hepatocellular carcinoma: a retrospective cohort study. Front Immunol. 2022;13:848387. 35300325 10.3389/fimmu.2022.848387 PMC8921060

[4] Kaibori M , Hiraoka A , Iida H , et al. Comparison of the new neo‐Glasgow prognostic score based on the albumin‐bilirubin grade with currently used nutritional indices for prognostic prediction following surgical resection of hepatocellular carcinoma: a multicenter retrospective study in Japan. Cancers (Basel). 2022;14(9):2091. 35565221 10.3390/cancers14092091 PMC9105166

[5] Yoo JJ , Chung GE , Lee JH , et al. Sub‐classification of advanced‐stage hepatocellular carcinoma: a cohort study including 612 patients treated with sorafenib. Cancer Res Treat. 2018;50(2):366‐373. 28521494 10.4143/crt.2017.126 PMC5912123

[6] Shinkawa H , Tanaka S , Takemura S , Ishihara T , Yamamoto K , Kubo S . Tumor size drives the prognosis after hepatic resection of solitary hepatocellular carcinoma without vascular invasion. J Gastrointest Surg. 2020;24(5):1040‐1048. 31197685 10.1007/s11605-019-04273-2

[7] Liang BY , Gu J , Xiong M , et al. Tumor size may influence the prognosis of solitary hepatocellular carcinoma patients with cirrhosis and without macrovascular invasion after hepatectomy. Sci Rep. 2021;11(1):16343. 34381132 10.1038/s41598-021-95835-5 PMC8357938

[8] Dai CY , Lin CY , Tsai PC , et al. Impact of tumor size on the prognosis of hepatocellular carcinoma in patients who underwent liver resection. J Chin Med Assoc. 2018;81(2):155‐163. 29217359 10.1016/j.jcma.2017.06.018

[9] Liu D , Song T . Changes in and challenges regarding the surgical treatment of hepatocellular carcinoma in China. Biosci Trends. 2021;15(3):142‐147. 33716267 10.5582/bst.2021.01083

[10] Carr BI , Guerra V . Features of massive hepatocellular carcinomas. Eur J Gastroenterol Hepatol. 2014;26(1):101‐108. 23863262 10.1097/MEG.0b013e3283644c49

[11] Xie LL , Sun CJ , Li XD , et al. Arterial embolization of massive hepatocellular carcinoma with lipiodol and gelatin sponge. Indian J Cancer. 2015;51(Suppl 2):e49‐e51. 25712844 10.4103/0019-509X.151990

[12] Ikai I , Kudo M , Arii S , et al. Report of the 18th follow‐up survey of primary liver cancer in Japan. Hepatol Res. 2010;40(11):1043‐1059. 34818831 10.1111/j.1872-034X.2010.00731.x

[13] Zhu SL , Chen J , Li H , Li LQ , Zhong JH . Efficacy of hepatic resection for huge (≥ 10 cm) hepatocellular carcinoma: good prognosis associated with the uninodular subtype. Int J Clin Exp Med. 2015;8(11):20581‐20588. 26884976 PMC4723821

[14] Chang YJ , Chung KP , Chang YJ , Chen LJ . Long‐term survival of patients undergoing liver resection for very large hepatocellular carcinomas. Br J Surg. 2016;103(11):1513‐1520. 27550624 10.1002/bjs.10196

[15] Mok KT , Wang BW , Lo GH , et al. Multimodality management of hepatocellular carcinoma larger than 10 cm. J Am Coll Surg. 2003;197(5):730‐738. 14585406 10.1016/j.jamcollsurg.2003.07.013

[16] Min YW , Lee JH , Gwak GY , et al. Long‐term survival after surgical resection for huge hepatocellular carcinoma: comparison with transarterial chemoembolization after propensity score matching. J Gastroenterol Hepatol. 2014;29(5):1043‐1048. 24863186 10.1111/jgh.12504

[17] Huang YH , Wu JC , Chen SC , et al. Survival benefit of transcatheter arterial chemoembolization in patients with hepatocellular carcinoma larger than 10 cm in diameter. Aliment Pharmacol Ther. 2006;23(1):129‐135. 16393290 10.1111/j.1365-2036.2006.02704.x

[18] Bhanu JS , Venkitaraman B , Palaniappan R , Ranganathan R , Seshadri RA , Mahajan V . Prognostic factors and survival outcomes of surgical resection of huge hepatocellular carcinomas. J Gastrointest Cancer. 2020;51(1):250‐253. 31054105 10.1007/s12029-019-00240-x

[19] Wang XH , Liu QB , Xiang CL , et al. Multi‐institutional validation of novel models for predicting the prognosis of patients with huge hepatocellular carcinoma. Int J Cancer. 2021;149(1):127‐138. 33586134 10.1002/ijc.33516

[20] Chen Z , Cai M , Wang X , et al. Two novel online nomograms for predicting the survival of individual patients undergoing partial hepatectomy for huge hepatocellular carcinoma. HPB (Oxford). 2021;23(8):1217‐1229. 33413992 10.1016/j.hpb.2020.12.002

[21] Marrero JA , Kulik LM , Sirlin CB , et al. Diagnosis, staging, and management of hepatocellular carcinoma: 2018 practice guidance by the American Association for the Study of Liver Diseases. Hepatology. 2018;68(2):723‐750. 29624699 10.1002/hep.29913

[22] Vogel A , Cervantes A , Chau I , et al. Hepatocellular carcinoma: ESMO clinical practice guidelines for diagnosis, treatment and follow‐up. Ann Oncol. 2018;29(Suppl 4):iv238‐iv255. 30285213 10.1093/annonc/mdy308

[23] European Association for the Study of the Liver . EASL clinical practice guidelines: management of hepatocellular carcinoma. J Hepatol. 2018;69(1):182‐236. 29628281 10.1016/j.jhep.2018.03.019

[24] Xie DY , Ren ZG , Zhou J , Fan J , Gao Q . 2019 Chinese clinical guidelines for the management of hepatocellular carcinoma: updates and insights. Hepatobiliary Surg Nutr. 2020;9(4):452‐463. 32832496 10.21037/hbsn-20-480 PMC7423548

[25] Fang Q , Xie QS , Chen JM , et al. Long‐term outcomes after hepatectomy of huge hepatocellular carcinoma: a single‐center experience in China. Hepatobiliary Pancreat Dis Int. 2019;18(6):532‐537. 31543313 10.1016/j.hbpd.2019.09.001

[26] Zhou YM , Li B , Xu DH , Yang JM . Safety and efficacy of partial hepatectomy for huge (≥10 cm) hepatocellular carcinoma: a systematic review. Med Sci Monit. 2011;17(3):RA76‐RA83. 21358616 10.12659/MSM.881443 PMC3524737

[27] Goh BK , Kam JH , Lee SY , et al. Significance of neutrophil‐to‐lymphocyte ratio, platelet‐to‐lymphocyte ratio and prognostic nutrition index as preoperative predictors of early mortality after liver resection for huge (≥10 cm) hepatocellular carcinoma. J Surg Oncol. 2016;113(6):621‐627. 26861568 10.1002/jso.24197

[28] Chen XP , Qiu FZ , Wu ZD , Zhang BX . Chinese experience with hepatectomy for huge hepatocellular carcinoma. Br J Surg. 2004;91(3):322‐326. 14991633 10.1002/bjs.4413

[29] Shrager B , Jibara GA , Tabrizian P , Schwartz ME , Labow DM , Hiotis S . Resection of large hepatocellular carcinoma (≥10 cm): a unique western perspective. J Surg Oncol. 2013;107(2):111‐117. 22903563 10.1002/jso.23246

[30] Lim C , Compagnon P , Sebagh M , et al. Hepatectomy for hepatocellular carcinoma larger than 10 cm: preoperative risk stratification to prevent futile surgery. HPB (Oxford). 2015;17(7):611‐623. 25980326 10.1111/hpb.12416 PMC4474509

[31] Jia C , Weng J , Qin Q , Chen Y , Huang X , Fu Y . Anatomic trisegmentectomy: an alternative treatment for huge or multiple hepatocellular carcinoma of right liver. Biomed Pharmacother. 2017;88:684‐688. 28152477 10.1016/j.biopha.2016.12.136

[32] Kabir T , Syn NL , Guo Y , Lim KI , Goh BKP . Laparoscopic liver resection for huge (≥10 cm) hepatocellular carcinoma: a coarsened exact‐matched single‐surgeon study. Surg Oncol. 2021;37:101569. 33839442 10.1016/j.suronc.2021.101569

[33] Xiang F , Liang X , Yang L , Liu X , Yan S . CT radiomics nomogram for the preoperative prediction of severe post‐hepatectomy liver failure in patients with huge (≥ 10 cm) hepatocellular carcinoma. World J Surg Oncol. 2021;19(1):344. 34895260 10.1186/s12957-021-02459-0 PMC8667454

[34] Zhu G , Wang W , Liu Q , et al. A real‐world study of prognosis of N0M0 hepatocellular carcinoma with hepatic resection based on SEER database. Gastroenterol Res Pract. 2020;2020:2357840. 32328093 10.1155/2020/2357840 PMC7152960

[35] Li X , Bi X , Zhao J , et al. A nomogram to predict prognosis after surgery for young patients with hepatocellular carcinoma. Transl Cancer Res. 2021;10(4):1773‐1786. 35116501 10.21037/tcr-20-3411 PMC8798826

[36] Ni X , Li D , Dai S , et al. Development and evaluation of nomograms to predict the cancer‐specific mortality and overall mortality of patients with hepatocellular carcinoma. Biomed Res Int. 2021;2021:1658403. 33860031 10.1155/2021/1658403 PMC8024067

[37] Zhou Y , Zhou X , Ma J , et al. Nomogram for predicting the prognosis of patients with hepatocellular carcinoma presenting with pulmonary metastasis. Cancer Manag Res. 2021;13:2083‐2094. 33688251 10.2147/CMAR.S296020 PMC7935331

[38] Nagai S , Yoshida A , Facciuto M , et al. Ischemia time impacts recurrence of hepatocellular carcinoma after liver transplantation. Hepatology. 2015;61(3):895‐904. 25099130 10.1002/hep.27358

[39] Zavaglia C , De Carlis L , Alberti AB , et al. Predictors of long‐term survival after liver transplantation for hepatocellular carcinoma. Am J Gastroenterol. 2005;100(12):2708‐2716. 16393224 10.1111/j.1572-0241.2005.00289.x

